# Evaluating Ki-67 and PR as prognostic indicators in CDK4/6 inhibitor treatment for metastatic breast cancer

**DOI:** 10.3332/ecancer.2025.1963

**Published:** 2025-08-12

**Authors:** María Florencia Illia, Giuliana Colucci, Angeles Ballester, Mariana Eiben, Fernando Paesani, Francisco Von Stecher, Máximo de la Vega, Florencia Perazzo, Pablo Mandó

**Affiliations:** 1 Clinical Oncology Department, Centro de Educación Médica e Investigaciones Clínicas “Norberto Quirno” (CEMIC), Buenos Aires CP 1431, Argentina; 2 Gynecology Department, Centro de Educación Médica e Investigaciones Clínicas “Norberto Quirno” (CEMIC), Buenos Aires CP 1431, Argentina; 3 Patology Department, Centro de Educación Médica e Investigaciones Clínicas “Norberto Quirno” (CEMIC), Buenos Aires CP 1431, Argentina

**Keywords:** breast cancer, CDK4/6 inhibitor, Ki-67, progesterone receptor

## Abstract

**Background:**

The treatment of choice as the first line for patients with metastatic breast cancer (MBC) who are hormone receptor-positive (HR+)/HER2-negative (HER2-) is the combination of endocrine therapy (ET) with cyclin-dependent kinase 4/6 inhibitors (CDK4/6i). Identifying prognostic or predictive factors of response could have important clinical implications. This study analysed the prognostic value of Ki-67 and progesterone receptor (PR) expression on progression-free survival (PFS) in this population.

**Methods:**

A retrospective cohort study was conducted in patients with HR+/HER2- MBC, who had received first-line treatment with CDK4/6i combined with ET. For the PFS analysis, the log-rank test was used and for the multivariate analysis, a Cox regression analysis was performed.

**Results:**

A total of 155 patients were analysed. Patients with PR values <20% showed a trend in univariate analysis towards shorter PFS, with a median of 20.7 months compared to those with a value ≥20%, with a median PFS of 33.0 months (*p* = 0.090). The Ki-67 value showed no statistically significant association with PFS. The prognostic role of PR was confirmed in the multivariate analysis with an HR of 0.59 (95% CI 0.36–0.98, *p* = 0.041) in patients with PR >20%.

**Conclusion:**

Patients with PR values <20% tended to have shorter PFS, unlike the Ki-67 value, which did not demonstrate an impact on PFS. This suggests a prognostic value of PR expression levels in this scenario.

## Introduction

Breast cancer is the most common malignant neoplasm and the leading cause of morbidity in women worldwide [1]. The majority of breast cancers, approximately 70%, are hormone receptor-positive (HR+) and HER2-negative (HER2-) [2]. In recent decades, there has been a notable improvement in the prognosis of patients with HR+/HER2- metastatic breast cancer (MBC), achieving a median overall survival (OS) of 57 months [3]. Currently, the treatment of choice in this scenario is the combination of endocrine therapy (ET) with cyclin-dependent kinase 4/6 inhibitors (CDK4/6i), approved for both first and second-line treatment [4]. Multiple phase III studies have demonstrated a benefit in progression-free survival (PFS) and OS with this treatment. There are three CDK4/6i approved and used in daily practice (ribociclib, palbociclib and abemaciclib), which have not been compared with each other in terms of efficacy, although only ribociclib and abemaciclib have shown an OS benefit [5–13].

Several studies have been conducted to identify clinical and/or pathological prognostic or predictive factors of response, with controversial results. Two meta-analyses, including more than 4,000 patients from major phase III studies on CDK4/6i, observed that the addition of CDK4/6i to hormonal treatment benefited all subgroups and several traditional prognostic factors such as disease site or progesterone receptor (PR) expression did not negatively impact PFS [14, 15]. However, other studies suggest that low PR expression and high proliferation index (Ki-67) are associated with poorer prognosis [16–18].

In Latin America, evidence on biomarkers for CDK4/6i response is scarce and fragmented. Only few studies have explored the role of PR and Ki67 but not in the scenario of CDK4/6i. Furthermore, these studies were retrospective, monocentric and limited by small sample sizes. Notably, no data exist from other Latin American countries, including Argentina, where differences in genetic ancestry, access to diagnostics and treatment patterns may influence results.

This study aims to address these gaps by evaluating the prognostic value of PR and Ki-67 in a well-characterised Argentine cohort of HR+/HER2− MBC patients treated with first-line CDK4/6i + ET. Our findings intend to clarify controversies from global trials while informing risk stratification in underrepresented Latin American populations with access difficulties to this treatment strategies.

## Objectives

The primary objective was to evaluate the prognostic value of PR and Ki-67 expression on PFS in patients with HR+/HER2- MBC who received first-line treatment with CDK4/6i combined with ET. Second, we assessed the impact on PFS of other factors such as tumour phenotype, the site of metastatic involvement and prior hormonal treatment in the adjuvant setting.

## Patients and methods

A retrospective cohort study was conducted on patients with MBC treated for their primary pathology at a center in Argentina. Patients were selected through non-probabilistic sampling from the breast cancer databases of the participating institution.

Patients included were those over 18 years old, diagnosed with HR+/HER2- MBC and undergoing first-line treatment with CDK4/6i combined with ET (tamoxifen + ovarian suppression or aromatase inhibitor) from 1 January 2018 to 15 August 2023. All patients had determinations of estrogen receptors (ER), PR, HER2 and Ki-67 by immunohistochemistry. In cases where these determinations were made on a metastasis biopsy, those results were used for analysis. For patients who did not undergo a biopsy for clinical reasons, the prognostic impact was evaluated using the results of the primary tumour.

Patients’ phenotypes were classified according to IHC results. Patients with positive ER expression, PR ≥ 20% and Ki-67 < 20% were classified as Luminal A-like, while patients with PR < 20% and/or Ki-67 ≥ 20% were classified as Luminal B-like. If the Ki-67 value was absent, patients were not classified into any group.

PFS was considered from the first date of CDK4/6i treatment until the evidence of progression was determined by the physician in the electronic medical record. OS was defined as the interval from the first date of CDK4/6i treatment until the date of death by any cause.

A specific electronic database was created for this purpose, and data were obtained from the patients’ electronic medical records. The data were coded to maintain anonymity and protect personal information. The project received approval from the Ethics, Teaching and Research Committee. Due to the study’s characteristics, informed consent was waived.

### Statistical analysis

Continuous variables were expressed as means (standard deviation) or medians (interquartile range) depending on the distribution, and discrete variables were expressed as binomial or as percentages. For the PFS analysis, a comparative evaluation of patients with or without the considered prognostic variable was performed using the log-rank test. Variables with a *p* ≤ 0.1 were considered for the construction of a multivariate model using Cox regression analysis. Differences were considered statistically significant with a *p* ≤ 0.05. Data were handled using RStudio software.

## Results

A total of 155 patients who received treatment with CDK4/6i combined with ET were included. The baseline characteristics of the population are described in Table 1. The median age at diagnosis was 55 years. A total of 32.3% of the patients presented with de novo metastasis. The majority were diagnosed with non-specific type (NST) breast cancer (61.9%) and luminal B phenotype (54.8%), with a median ER expression of 90%, PR of 60% and Ki-67 of 20%. The sites of disease involvement were exclusively bone in 38.3% of cases and mixed involvement (visceral and bone or lymph node) in 35.1%.

The previous treatments in the adjuvant setting are presented in Table 2, highlighting that 48.4% of the patients received chemotherapy, 52.9% received radiotherapy and 63.8% received ET based on aromatase inhibitors (51.5%) and tamoxifen (39.4%).

With a median follow-up of 33.5 months (IQR 14.1–51.2), the median PFS for the entire population was 24.3 months.

### Analysis of variables associated with PFS

**Figure 1.**
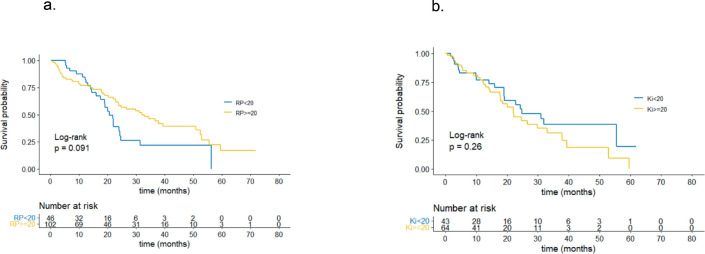
(a): PFS by PR. (b): PFS by Ki67.

According to the univariate analysis, patients with PR values <20% showed a non-significant trend towards shorter PFS with a median of 20.7 months, compared to patients with PR values ≥20%, who had a median PFS of 33.0 months (*p* = 0.091), as shown in Figure 1a. The Ki-67 value was not associated with significant differences in PFS. Patients with a Ki-67 value ≥20% had a median PFS of 22.0 months compared to those with a Ki-67 value <20%, who had a median PFS of 24.8 months (*p* = 0.26). These findings are represented in Figure 1b.

**Figure 2.**
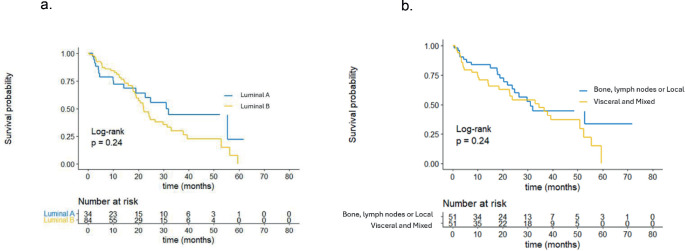
(a): PFS by phenotype. (b): PFS by site of metastasis.

The breast cancer phenotype, classified by IHC, did not show statistically significant differences in PFS between groups. The median PFS was 32.0 months for patients with Luminal A-like breast cancer and 22.0 months for patients with Luminal B-like breast cancer (*p* = 0.24), as depicted in Figure 2a. Similarly, the site of metastasis did not demonstrate significant differences in PFS. According to the results, patients with bone, lymph node or locoregional involvement had a median PFS of 34.5 months compared to those with visceral or mixed involvement, who had a median PFS of 31.1 months (*p* = 0.24). These results are illustrated in Figure 2b.

**Figure 3.**
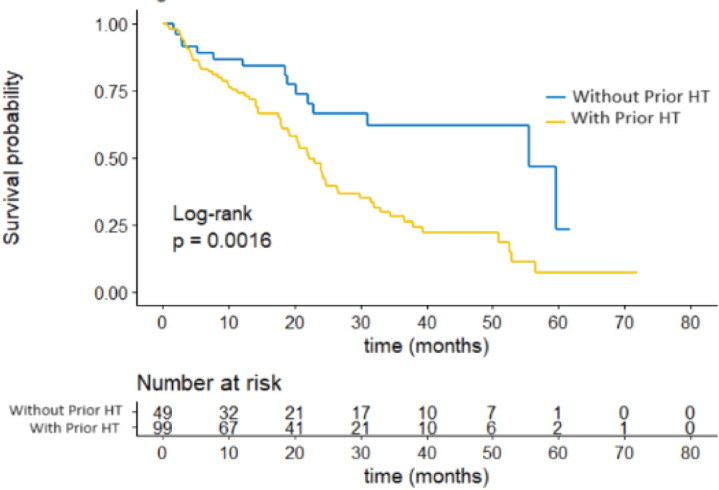
PFS by hormonal treatment.

Regarding previous treatments, it was observed that the use of adjuvant ET was associated with shorter PFS, with a median of 22.1 months compared to those who had not received it previously, with a median PFS of 55.5 months, a statistically significant difference (*p* = 0.0016). These results are presented in Figure 3.

### Multivariate analysis

**Figure 4.**
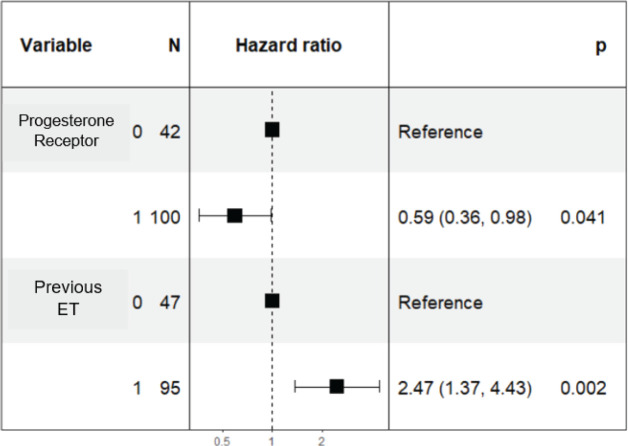
Forest plot of multivariate analysis of PR and prior hormone therapy.

In the multivariate analysis, it was determined that a PR value ≥20% independently predicts better prognosis adjusted for other variables and is associated with longer PFS with an HR of 0.59 (95% CI 0.36–0.98; *p* = 0.04) (Figure 4). Additionally, prior ET maintains its prognostic role and is associated with poorer PFS with an HR of 2.47 (95% CI 1.37–4.43; *p* = 0.002).

## Discussion

In our study, we analysed the prognostic value of PR and Ki-67 expression levels in patients with HR+/HER2- MBC undergoing first-line treatment with CDK4/6i combined with hormonal therapy, demonstrating that PR expression level serves as a prognostic factor, while Ki-67 expression did not show significant prognostic value in this setting. Additionally, we observed that prior exposure to hormonal therapy in the adjuvant setting also impacts therapeutic outcomes in the first-line treatment of metastatic disease.

These findings contribute to the growing body of evidence supporting the clinical utility of PR status assessment, particularly in the context of CDK4/6i therapy. Our results align with recent studies by Guliyev *et al* [19] and Jia *et al* [20], who similarly found that PR-negative status was associated with poorer outcomes in patients treated with CDK4/6i, suggesting this may be a consistent finding across diverse populations. Moreover, results are consistent with a retrospective analysis that included patients from the Phase III MONARCH2 and MONARCH3 studies, where negative PR status, high histological grade, liver metastases or a treatment-free interval from the end of adjuvant ET <36 months were associated with poorer prognosis. It was observed that these patient subgroups benefited most from the addition of CDK4/6i to ET [21]. Low PR expression could directly relate to sensitivity to hormonal treatments or even serve as a surrogate for molecular subtypes with poorer prognosis. In Latin American populations, there are two published studies from Mexico mentioning that luminal tumours with low PR expression exhibit characteristics of poorer prognosis and variable response to ET. They also discuss whether low PR expression could serve as an independent prognostic marker, as its absence is associated with more aggressive tumours and a worse prognosis [22, 23]. Nevertheless, none of these studies analyses its impact in the context of CDK4/6i.

The lack of prognostic significance for Ki-67 in our study contrasts with some previous reports, possibly reflecting the known challenges in Ki-67 assessment standardisation across institutions [24]. Small retrospective studies investigating the prognostic role of Ki-67 expression levels in advanced disease treated with first- or second-line CDK4/6i combined with ET versus ET alone observed that, with a cut-off value of ≥20%, Ki-67 was associated with a poorer prognosis [25]. This discrepancy highlights the need for caution when interpreting biomarker results, particularly in single-center studies with potential limitations in statistical power.

The recognition of prognostic factors for treatment outcomes in this scenario has significant clinical implications. It may allow a better selection of the subgroup of patients who would benefit most from this therapeutic strategy, as well as facilitate a more personalised approach for each patient. The recent SOLTI-1801 CDK-PREDICT study provides important context for our findings, demonstrating that intrinsic molecular subtypes may influence CDK4/6i efficacy, which could partially explain the variable results observed across different biomarker studies [26]. Nevertheless, access to molecular platforms determining intrinsic subtypes remain extremely infrequent in limited resource settings such as Latin America. Even more, it is important to highlight that these factors are prognostic and not predictive, as existing evidence indicates that all patients benefit from the addition of CDK4/6i to hormonal treatment, although not all subgroups experience the same PFS.

In this study, other clinical variables were also analysed as potential prognostic factors, such as the site of metastatic involvement, phenotype and prior hormone therapy use. Our observation that prior adjuvant hormonal therapy exposure negatively impacts PFS in the metastatic setting raises important questions about treatment resistance mechanisms. Specific resistance mechanisms, such as pRB or FAT1 mutations, have been described for CDK4/6i, suggesting a potential correlation with hormone therapy [27]. Our finding warrants further investigation, particularly in light of emerging therapies such as elacestrant that target ESR1 mutations, which may be more prevalent in this patient population, suggesting the possibility of combining them with CDK4/6i [28].

Several limitations must be acknowledged when interpreting these results. The retrospective nature of our study introduces potential biases in data collection and patient selection. While our sample size is comparable to similar single-center studies, we recognise the possibility of underpowered analyses for some subgroup comparisons. The absence of centralised pathology review may have introduced variability in biomarker assessment, though this reflects real-world clinical practice conditions. These limitations underscore the need for prospective validation of our findings in larger, multicenter studies with standardised biomarker evaluation protocols.

## Conclusion

In conclusion, our study supports the prognostic value of PR expression in HR+/HER2- MBC patients treated with CDK4/6i, while highlighting the ongoing challenges in establishing reliable biomarkers for this treatment modality. These findings contribute to the growing evidence from international studies while providing specific data from our regional population. Future research should focus on the prospective validation of these results and exploration of the underlying biological mechanisms linking PR expression to treatment outcomes. Such efforts will be crucial for advancing personalised treatment strategies in this patient population.

## Conflicts of interest

The authors report no conflicts of interest in this work.

## Funding

This research received no specific grant from any funding agency in the public, commercial or not-for-profit sectors.

## Figures and Tables

**Table 1.** General characteristics of the population.

Patients (*n*)	155
Age at diagnosis^*^	55 (43–66.25)
Stage at diagnosis (%)	
- I	22 (14.2)
- II	36 (23.2)
- III	29 (18.7)
- IV	50 (32.3)
- Unknown	18 (11.6)
- De novo (%)	50 (32.3)
Histology (%)	
- NST (No special type)	96 (61.9)
- Lobular	22 (14.2)
- Ductolobular	6 (3.9)
- Mucinous	2 (1.3)
- Papillary	2 (1.3)
- Micropapillary	1 (0.6)
- Unknown	26 (16.8)
- ER expression^*^	90 (80–100)
- PR expression^*^	60 (10–80)
- Ki67 expression^*^	20 (12–35)
Phenotype	
- Luminal A-like	34 (21.9)
- Luminal B-like	85 (54.8)
- Undefined	36 (23.2)
Sites involved by disease	
- Local - unresectable	3 (1.9)
- Only lymph nodes	8 (5.2)
- Only bone	59 (38.1)
- Visceral	30 (19.3)
- Mixed	55 (35.5)

* Median (IQR)

**Table 2.** Previous treatments in relapsed patients (% are based on the relapsed population *n* = 105).

Patients (*n*)	105
Adjuvant chemotherapy (%)	75 (71.4)
Type of chemotherapy	
- Anthracyclines	8 (10.6)
- Anthracyclines + Taxanes	54 (72.0)
- No anthracyclines or taxanes	7 (9.3)
- Taxanes	4 (5.3)
- Unknown	2 (2.7)
- Radiation therapy (%)	82 (78)
- Hormone therapy (%)	99 (94.2)
Type of hormone therapy (%)	
- Aromatase inhibitor	47 (47.5)
- Aromatase inhibitor + Ovarian suppression	4 (4.0)
- Tamoxifen	35 (35.4)
- Tamoxifen + Ovarian suppression	4 (4.0)
- Unknown	9 (9.1)
